# Editorial: Epidemiology and Control of Notifiable Animal Diseases

**DOI:** 10.3389/fvets.2019.00043

**Published:** 2019-02-26

**Authors:** Julio Alvarez, Douwe Bakker, Javier Bezos

**Affiliations:** ^1^Department of Veterinary Population Medicine, College of Veterinary Medicine, University of Minnesota, Saint Paul, MN, United States; ^2^Centro de Vigilancia Sanitaria Veterinaria VISAVET, Universidad Complutense, Madrid, Spain; ^3^Departamento de Sanidad Animal, Facultad de Veterinaria, Universidad Complutense, Madrid, Spain; ^4^Independent Researcher, Lelystad, Netherlands

**Keywords:** control, notifiable disease, tuberculosis, surveillance, risk assessment, modeling

## Introduction

There are a number of criteria by which an animal disease is classified as notifiable; the most important are typically related with its potential to spread internationally, as well as its impact on the health of domestic livestock, wildlife and, not the least, on human health (1). Because of the above, surveillance, early detection, control, and eradication of these diseases is of critical importance for countries in order to maintain or improve their animal health status. This requires the collaboration of all stakeholders involved (e.g., animal health authorities, livestock industry, and veterinary research institutions). The ability to prevent or respond adequately to the novel introduction of a notifiable disease into a herd, region or country, to control its spread and eventually accomplish its eradication requires the availability of adequate diagnostic tests for a preferably early detection of infected animals, an adequate knowledge on its epidemiology including the potential routes of transmission within or between herds and, ideally, the existence of vaccines to avoid disease dissemination. Altogether this can help to ensure an optimal allocation of the existing resources to minimize the likelihood of a disease outbreak, as well as its potential spread and negative impact. This research topic includes a variety of articles focusing on different aspects of surveillance, control, and eradication of diseases of critical importance for livestock, including cattle, swine, and wildlife, in an attempt to provide an overview of the current situation in different countries of the world.

## Disease Detection

One of the requirements for a disease to become notifiable is the existence of a reliable means of detection and diagnosis that allow a precise case definition (1), something often challenging in the case of livestock diseases. A typical example of such a challenge is bovine tuberculosis (bTB): despite major efforts invested in its control and eradication, the disease is still present in many countries worldwide, due in part to the limitations of the current diagnostic tests and their application. Three articles of this research topic either review or evaluate the performance of the two tests most commonly used for its diagnosis, the tuberculin skin test and the *in vitro* interferon-gamma release assay (IGRA) (Good et al.; Keck et al.; de la Cruz et al.). Good et al. provide an overview of the past and present use of the tuberculin skin test for bTB detection since its first applications by the end of the nineteenth century and describe the current challenges for the development of improved alternative tests. The other two articles provide evidences of the usefulness of the most widely used tool for bTB diagnosis other than the tuberculin test, the IGRA as an ancillary test for maximizing diagnostic sensitivity. Keck et al. demonstrate how the introduction of the IGRA has helped to decrease bTB prevalence in a specific cattle subpopulation in which the tuberculin test is difficult to perform, bullfighting cattle, in France. Finally, de la Cruz et al. compared two different commercial IGRA kits that have been used in Spain for bTB detection, finding large differences in terms of their sensitivity, and suggesting that their usefulness could be optimized by adjusting the cut-off values as recommended by the manufacturer.

## Risk Assessment

Introduction of a non-endemic disease into a region can have catastrophic consequences. These consequences can be particularly dramatic when/if the disease has a high impact on animal health, the need for its surveillance detection complicates the trade of animals and animal products, wildlife species can subsequently become reservoirs of the disease, and tools like vaccination to reduce the susceptible population are not available. Brown and Bevins coauthor two studies that provide a detailed review of the epidemiology and risk of introduction and establishment into the United States of America of two of the most important swine diseases that are currently expanding their geographical range: classical swine fever (Brown and Bevins) and African swine fever (Brown and Bevins).

## Disease Modeling

Increased knowledge of the epidemiology of a well-established disease can also be of great use, since it will assist in the improvement of current control programmes. This knowledge often depends on the population under study, and therefore different factors may drive disease distribution in different settings. The use of explanatory, predictive and spread models can help to identify those factors and assess their impact. Clegg et al. studied the factors associated with larger bTB outbreaks in cattle farms in Ireland in an attempt to identify parameters that would allow the implementation of better targeted policy measures. Haredasht et al. used slaughter surveillance data to characterize the temporal trends in cattle condemnation in the US in general and California in particular in order to develop tools for detection of changes in carcass condemnation rates that would help to design prevention and mitigation strategies to minimize its impact. Mourant et al. developed a compartmental disease spread model to evaluate the potential impact of mitigation strategies such as vaccination, and validated it using historical data on rinderpest outbreaks as an example.

## Experimental Studies

In certain cases, however, data derived from simulation models or observational studies may not be sufficient to draw conclusions on the efficacy of preventive and control measures, and additional experimental data are required. Again bTB is a perfect example of such a situation due to its complex epidemiology and chronic nature, which makes predicting and interpreting field data particularly challenging. Gormley and Corner review how the careful evaluation of data from naturally infected badgers can serve as the basis to design experimental infection studies shown to be essential for the evaluation of diagnostic assays and to measure vaccine efficacy in this species. Finally, Serrano et al. hypothesized about the potential beneficial effect of an inactivated paratuberculosis vaccine on bTB progression in calves using data generated through an experimental *M. bovis* challenge.

## Author Contributions

All authors listed have made a substantial, direct and intellectual contribution to the work, and approved it for publication.

### Conflict of Interest Statement

The authors declare that the research was conducted in the absence of any commercial or financial relationships that could be construed as a potential conflict of interest.
